# Socio-economic inequality and HIV in South Africa

**DOI:** 10.1186/1471-2458-13-1037

**Published:** 2013-11-04

**Authors:** Njeri Wabiri, Negussie Taffa

**Affiliations:** 1 Epidemiology and Strategic Information Unit, Human Sciences Research Council, Private Bag X41, Pretoria 0001, Gauteng, South Africa; 2 United States Center for Disease Control and Prevention (CDC), QED GrS2oup LLC, Windhoek, Namibia

## Abstract

**Background:**

The linkage between the socio-economic inequality and HIV outcomes was analysed using data from a population-based household survey that employed multistage-stratified sampling. The goal is to help refocus attention on how HIV is linked to inequalities.

**Methods:**

A socio-economic index (SEI) score, derived using Multiple Correspondence Analysis of measures of ownership of durable assets, was used to generate three SEI groups: Low (poorest), Middle, and Upper (no so poor). Distribution of HIV outcomes (i.e. HIV prevalence, access to HIV/AIDS information, level of stigma towards HIV/AIDS, perceived HIV risk and sexual behaviour) across the SEI groups, and other background characteristics was assessed using weighted data. Univariate and multivariate logistic regression was used to assess the covariates of the HIV outcomes across the socio-economic groups. The study sample include 14,384 adults 15 years and older.

**Results:**

More women (57.5%) than men (42.3%) were found in the poor SEI [P<0.001]. HIV prevalence was highest among the poor (20.8%) followed by those in the middle (15.9%) and those in the upper SEI (4.6%) [P<0.001]. It was also highest among women compared to men (19.7% versus 11.4% respectively) and among black Africans (20.2%) compared to other races [P<0.001]. Individuals in the upper SEI reported higher frequency of HIV testing (59.3%) compared to the low SEI (47.7%) [P< 0.001]. Only 20.5% of those in poor SEI had “good access to HIV/AIDS information” compared to 79.5% in the upper SEI (P<0.001). A higher percentage of the poor had a stigmatizing attitude towards HIV/AIDS (45.6%) compared to those in the upper SEI (34.8%) [P< 0.001]. There was a high personal HIV risk perception among the poor (40.0%) and it declined significantly to 10.9% in the upper SEI.

**Conclusions:**

Our findings underline the disproportionate burden of HIV disease and HIV fear among the poor and vulnerable in South Africa. The poor are further disadvantaged by lack of access to HIV information and HIV/AIDS services such as testing for HIV infection. There is a compelling urgency for the national HIV/AIDS response to maximizing program focus for the poor particularly women.

## Background

The debate on the link between poverty and HIV infection in sub-Saharan Africa has continued for almost two decades without definite consensus. A large body of literature in the early years of the HIV epidemic indicated that relative wealth was associated with a higher risk of HIV infection [1,2]. Owing to the relative abundance of disposable income, individuals and households in the higher income groups were more likely to be engaged in risky multiple concurrent sexual partnerships. As the epidemic matured, those in the poorer income brackets began to become equally at risk of HIV infection, mainly due to the expansion of sexual networks and also due to the increasing transactional nature of sex. During the second decade of HIV epidemic, the lost economic opportunities and cost of caring for the sick and orphaned became severe among poorer households and communities. This socio-economic impact of HIV/AIDS led to HIV becoming strongly associated with poverty [3,4]. These assertions were, however, context specific. Around 2005, it emerged that socio-economic inequality and vulnerability, rather than just poverty were most strongly associated with HIV occurrence in sub-Saharan Africa. Piot, Greener, & Russell (2007) and Temah (2008), reported that African countries with greatest Gini Coefficient Index were hardest hit by the epidemic, and most of these countries were found in the Southern African region reaffirming that HIV/AIDS is a disease of inequality rather than of poverty.

One reason behind the debate is rooted in methodological shortcomings to measure income and poverty at the individual and household levels. Population-based Demographic and Health Surveys (DHS), AIDS Impact Surveys (AIS) and other cluster sample surveys use household ownership of items such as radios, refrigerators, phones and the availability of social amenities such as water, electricity and toilets to indicate levels of poverty. It is now believed that possession of these assets and amenities inadequately discriminates poor and non-poor households [5,6].Wealth and social status in rural Africa are still expressed in land, cattle and agricultural ownership, despite a more urban lifestyle becoming common in African populations.

The cross-sectional nature of data sources that were used to link poverty and HIV infection is another source of controversy because such studies are faced with the dilemma of inferring causality between poverty and HIV infection. Nevertheless, derived socio-economic status or inequality measures are widely used in epidemiological research and are crucial not only for studies focusing on the social determinants of health, but also for the vast majority of observational health research [7].

Previous studies on poverty and HIV infection in South Africa have revealed mixed results. Booysen and Summerton [8] analysed the 1998 DHS data and found that socio-economic inequality, including gender, did not have significant association with HIV infection and sexual risk behaviour. Steinberg et.al [9] reported that households worst hit by HIV were also least served by basic social services such as water and sanitation showing that poor people are most affected by HIV/AIDS in South Africa. However, longitudinal surveillance data from rural Kwazulu-Natal indicated differences in household educational attainment to be a much stronger factor for HIV acquisition than income and expenditure [10].

Using the 2008 South African National HIV prevalence, incidence, behaviour and communication survey [11], this study examines the association of socio-economic inequality as measured by the household asset index, with key HIV-related outcomes such as HIV prevalence, HIV risk perception, sexual behaviour and utilization of HIV testing services. The study hopes to clarify the above mixed results reported on the link between socio-economic inequality and HIV in South Africa.

### Data source

The study used data from the 2008 South African National HIV prevalence, incidence, behaviour and communication survey [11]. This is a cross-sectional population-based household survey conducted every 3–4 years using a multi-stage stratified sampling approach (by province, geo-type and predominant racial groups). Sampling frames were based on enumeration areas (EA) used in the national census, with updates to reflect changes in the socio-demographic profile of the country since the last census in 2001. A total of 1000 EAs were selected as the primary sampling units, 15 households within EAs formed the secondary sampling unit and four eligible individuals selected within households formed the final sampling unit. All household members in the selected households were eligible to participate, including those living in hostels. People staying in educational institutions, old-age homes, hospitals and uniformed-service barracks, as well as homeless people, were excluded from the survey. A household was defined as a group of people living, cooking and eating together.

Dried blood spot (DBS) specimens were used for HIV antibody testing. An algorithm of three HIV enzyme immunoassays was used to test for HIV antibodies [12]. Full details of the survey methodology, including sample weighting, fieldwork procedures and quality control measures and ethical approval^a^ are described elsewhere [6,12].

## Methods

### Deriving study measures

Based on the multistage stratified sampling described above, this study draws on data collected from adults aged 15 years and above. Data are drawn from three face-to-face questionnaires: a household-level questionnaire; a youth aged 15–24 years questionnaire; and an adults aged 25 years and above questionnaire.

The socio-economic index (SEI) measures were derived from 32 items, in the household questionnaires, related to measures of household-living standards, such as infrastructure and housing characteristics (source of drinking water, access to electricity, main source of energy for cooking, and type of toilet used) and household ownership of durable assets (presence of a working refrigerator, radio, television, cell phone and landline phone). Quantiles were generated using the multiple correspondence analysis (MCA) [6,11]. Other studies that have used MCA to generate socio-economic index measures included the works of:- Asselin and Anh [13] in Vietnam; Ki et.al in Senegal [14]; Ndjanyou [15] and Njong [16] both for the Cameroon case. Booysen et. al utilised MCA to construct wealth indices for seven sub-Saharan African countries [17], while Cleary et.al used MCA to generate socio-economic status in assessing equity in the use of antiretroviral treatment in the public health care system in urban South Africa [18].

Three socio-economic index groups were used instead of the more widely used five groups due to the skewed distribution of the quintiles; the 5th quintile had only 0.6% of the total adults which meant that the frequency was too low frequency for meaningful analysis. Also the socio-economic class differences in the rural communities are narrow because of similar income generation activities at that level [19]. Hence, it was more realistic to use three socio-economic index groups to differentiate the households.

The Chronbach alpha coefficient for the resulting SEI was 0.7726. The large positive MCA weight of 1.067 for possession of a fixed telephone line (see Additional file 1: Table S1), was a notable sign of high socio-economic status. Absence of toilet (MCA weight of −3.190) and electricity (MCA weight of −3,061) were significantly associated with low socio-economic index or status. The working telephone (Std.Dev. of 0.463), sanitary services (Flush toilet) (Std.Dev of 0.467), and access to electricity (Std.Dev. of 0.412) also confirms that they are important in differentiating SEI scores among the household (See Additional file 1: Table S1).

A stigma score was constructed using factor analysis of five variables: ‘would you buy food from a shopkeeper who lives with HIV’, ‘care for HIV infected family member’, ‘disclose one’s HIV status with at least one family member’ and ‘relate to a teacher who lives with HIV’. The reliability and internal consistency of the resulting index was assessed using Chronbach alpha score and had a value of 0.6005. All the five variables had high correlation with the stigma score.

The information access score to estimate the level of access to HIV/AIDS information was constructed using factor analysis of five variables namely; frequency of use of TV, radio, newspaper, magazine and internet assuming that they constitute important sources of information on HIV/AIDS. The new variable on information access had a reliability index- Chronbach alpha score of 0.6497, showing that the variables used were consistent in explaining the information access construct.

HIV risk perception was measured from a personal risk assessment scale ranging between 1 and 4 (1 being low risk and 4 being high HIV risk perception). The survey respondents were asked to rate themselves on the risk of becoming infected with HIV based on four choices: - ‘l will definitely not get infected’; ‘I probably won’t get infected’; ‘I’m probably going to get infected’; and ‘I’m definitely going to get infected with HIV’. Response options such as I’m probably going to get infected; and I’m definitely going to get infected with HIV, were arbitrarily taken to imply high risk perception.

### Descriptive and regression analysis

Analysis was done in Stata version 11.0 (College Station, Texas, United States), taking into account the complex multilevel sampling design and participant non-response. STATA software (svy) commands were used to obtain the estimates of proportions and confidence intervals (95% CI). Summary indices for descriptive analysis are weighted^b^ percentages, and un-weighted counts are provided. In invariable analysis, the distribution of the study outcomes- HIV testing, HIV risk Perception and HIV prevalence- across population groups were compared using the Rao-Scott F statistic to determine *P* values [20]. Multivariate logistic regression analysis, using backward fitting, was used to identify factors associated with HIV testing, HIV prevalence and HIV risk Perception. The independent variables include socio-economic index, education, stigma score, information access score and selected background characteristics. Clustering was not accounted for given that the large number of primary sampling units (1000) in the study is comparable to respondent number, thus diminishing such effects.

## Results

Of the 15,000 households sampled, only 13,440 (89.3%) were occupied; 80.8% of whom were interviewed (10,856/13,440). Non-response was largely due to refusal (9.3%, 1252/13,440) or no household member at home after four repeat visits (7.0%, 946/ 13,440). The study sample N = 14,384 of adults 15–64 years, out of the 23,112 cases in the survey.

### Descriptive analysis results

#### Background characteristics across the socio-economic index (SEI)

Overall, 40.1% of the 14,384 adults (15–64 years) fall in the poor SEI group, 42.5% in middle and remaining 17.4% in upper SEI [P < 0.001] (Table 1). More women (57.5%) compared to men (42.5%) were found in the poor SEI group [P < 0.001]. All but three provinces (Gauteng 35.3%, Western Cape 22.2% and Kwazulu-Natal 17.9%), had less than 10% of respondents who belonged to the upper (not-so-poor) SEI group [P < 0.001]. Limpopo (18.8%), Eastern Cape (18.8%) and Kwazulu-Natal (23.0%) had the largest percentage of respondents belonging to the poor SEI [P < 0.001]. The largest percentage of respondents in poor SEI (57.5%) lived in the rural tribal land (or rural formal settlement) [P < 0.001]. More than 30% of respondents in poor SEI were urban residents, equally distributed in the formal and informal settlementt [P > 0.05]. More than 70% of those in the middle and 92% of the upper SEI group lived in formal urban settlements (P < 0.001). Eighty four per cent of respondents in the poor SEI had no formal education or completed only up to primary level compared to 31% in the upper SEI group [P < 0.001]. On the other hand, 68.2% of those in upper SEI and 36.3% of the middle SEI respectively had completed matric exam or tertiary education. Over 95% of people in the poor SEI group were black Africans while other races formed close to 47% of those belonging to the upper SEI [P < 001.

**Table 1:** Distribution of Socio-economic index (SEI) among adults (15–65 years) by selected background characteristics

**Characteristic**	**Socio-economic index (SEI) groups**		
	**Poor(low)**	**Middle**	**Upper**
	**% [95% CI]**	**% [95% CI]**	**% [95% CI]**
Sex***			
Male	42.3[40.2,44.4]	45.5[43.3,47.8]	45.1[41.6,48.6]
Female	57.7[55.6,59.8]	54.5[52.2,56.7]	54.9[51.4,58.4]
	*N = 4308*	*N = 5067*	*N = 2892*
Age (years)***			
15-24	*37.1[35.6,38.6]*	*31.3[29.9,32.8]*	*25.1[23.2,27.2]*
25-49	*48.8[46.9,50.8}*	*55.8[53.7,57.8]*	*54.2[51.2,57.2]*
50+	*14.1[12.9,15.4]*	*12.9[11.614.3]*	*20.6[18.4,23.0]*
	*N = 4308*	*N = 5067*	*N = 2892*
Race***			
African	96.2[95.2,97.0]	78.3[75.0,81.3]	35.7[30.0,41.8]
White	0.5[0.3,1.0]	8.0[6.4,9.8]	35.8[30.8,41.1]
Coloured	3.2[2.5,4.1]	11.5[9.7,13.7]	17.9[15.0,21.3]
Indian	0.1[0.0,0.1]	2.2[1.3,3.6]	10.6[8.5,13.0]
	*N = 4300*	*N = 5060*	*N = 2881*
Province***			
Western Cape	4.3[3.2,5.8]	13.8[11.4,16.6]	22.4[19.2,26.0]
Eastern Cape	18.8[15.5,22.6]	8.4[6.4,11.0]	9.6[6.8,13.3]
Northern Cape	1.4[1.1,1.8]	2.4[1.9,2.9]	2.8[2.1,3.7]
Free State	4.9[3.9,6.2]	8.2[6.3,10.8]	3.7[2.6,5.4]
KwaZulu-Natal	23.0[18.9,27.6]	18.1[14.6,22.3]	17.9[14.5,22.0]
North West	10.6[8.5,13.1]	7.8[6.2,9.6]	3.8[2.6,5.5]
Gauteng	9.0[6.9,11.7]	28.7[23.3,34.8]	35.3[30.0,41.0]
Mpumalanga	9.3[7.4,11.5]	6.7[5.0,9.0]	2.9[1.8,4.5]
Limpopo	18.8[15.9,22.0]	5.9[4.4,7.8]	1.8[1.1,2.7]
	*N = 4308*	*N = 5067*	*N = 2892*
Geographical location***			
Urban formal	16.6[13.5,20.4]	72.9[68.4,77.1]	92.4[88.8,94.9]
Urban informal	16.0[12.9,19.8]	7.8[5.6,10.8]	7.8[0.3,1.4]
Rural informal	57.5[52.5,62.4]	14.6[11.7,18.2]	1.9[0.7,5.0]
Rural formal	9.8[7.6,12.5]	4.6[3.3,6.3]	5.1[3.2,8.0]
	*N = 4308.0*	*N = 5067.0*	*N = 2892.0*
Education***			
No schooling	8.0[6.9,9.2]	2.3[1.8,3.1]	0.9[0.5,1.7]
Less than matric	75.5[73.5,77.4]	56.0[53.5,58.5]	31.0[27.7,34.5]
Matric	13.7[12.2,15.3]	30.5[28.3,32.8]	41.1[38.1,44.1]
Above Matric	2.8[2.1,3.8]	11.1[9.5,13.0]	27.1[24.0,30.4]
	*N = 4084*	*N = 4882*	*N = 2792*
Gross income before tax per annum***			
No Income	35.8[31.6,40.2]	18.0[15.1,21.2]	12.6[10.1,15.5]
< 6000	23.7[20.4,27.2]	14.0[11.8,16.5]	5.2[3.8,7.1]
6000-24000	27.4[24.3,30.9]	25.5[22.7,28.6]	16.4[13.2,20.1]
24000-96000	11.4[9.3,13.9]	29.7[26.7,33.0]	30.9[26.9,35.2]
>96000	1.7[1.0,3.1]	12.8[10.6,15.3]	34.9[30.9,39.2]
	*N = 1518*	*N = 2551*	*N = 1781*

***Significant at 0.1%.

#### HIV testing and prevalence across socio-economic index groups

Respondents in upper SEI reported higher percentage of HIV testing (59.3%) in the past followed by those in the middle (56.6%) and the poor (47.7%) respectively [P < 0.001].Although men generally reported testing for HIV infection less frequently than women, this difference was more substantial among the poor-a difference of 24.4% compared to a difference of 11.7% between men and women who were tested for HIV infection in the upper SEI group. Among the poor SEI group, only 33.6% of men reported HIV testing compared to 58% women [P < 0.001]. The rate of HIV testing was 52.8% and 64.5% for men and women respectively in the upper SEI [P < 0.001]. Other results (not shown) indicate that HIV testing was higher among whites (66.8%) although more than 50% of all other races also reported having ever been tested [P < 0.001].

HIV testing reports showed less disparity by major geographic locations except in rural informal (tribal) areas. On average, 56.0% of people in urban and formal rural areas reported to have been tested for HIV infection compared to 46.3% for those living in urban informal [P < 0.001]. Majority of respondents in the upper SEI did not receive HIV test from public health facilities. Accordingly, 89.0% of the poor, 64.5% of those in the middle and only 31.1% of the upper SEI group ever sought HIV test from government hospital or clinic [P < 0.001]. More than 97.0% of those who sought HIV test in public facilities expressed satisfaction in the service across all socio-economic index groups.

HIV prevalence was highest among the poor (20.8%) compared to those in the middle (15.9%) and upper SEI (4.6%) respectively [P < 0.001] (Table 2). HIV prevalence was significantly higher among women than men (19.7% versus 11.4% respectively); among black Africans (20.3%) compared to other races (3.4% among coloured and <1.0% among whites); and among urban informal settlement residents (28.5%) compared to those living in rural areas (18.2%) or urban formal areas (12.7%). Among the poor, HIV prevalence was almost twice as high among women compared to men (25.4% versus 14.0%) (Table 2). It was 5 times higher among black Africans when compared to coloured people (21.5% versus 4.4%), but 20 times higher when compared to whites or Indians who had almost no HIV positive test results [P < 0.001]. Similar patterns of race and gender differentials were seen when HIV prevalence data was examined among those in the middle and upper SEI groups. HIV prevalence followed the national pattern of high peak rate among 29–40 year olds across all the three socio-economic groups, but maintained socio-economic gradient of being highest among the poor and lowest among the upper SEI group (i.e. 30.5%, 22.3% and 6.4% respectively).

**Table 2:** HIV prevalence, HIV testing, HIV risk perception, HIV information access and HIV stigma among men and women (15-65 years) across social economic index groups

**Social economic index groups**	**HIV prevalence**		**HIV testing**		**HIV risk perception (High)**		**HIV information score (Low access)**		**HIV stigma score (High stigma)**	
	**n**	**% [95% CI]**	**n**	**% [95% CI]**	**n**	**% [95% CI]**	**n**	**% [95% CI]**	**n**	**% [95% CI]**
Overall***										
Poor	3470	20.8[18.8,22.9]	4078	47.7[45.6,49.9]	4063	40.0[37.9,42.1]	4051	79.5[77.5,81.5]	4066	45.6[43.2,48.0]
Middle	3981	15.9[14.0,18.1]	4877	56.6[54.2,59.0]	4873	26.0[23.7,28.5]	4859	37.4[35.3,39.6]	4865	34.4[31.9,37.0]
Upper	2110	4.6[2.6,7.9]	2789	59.3[56.3,62.2]	2789	10.9[8.5,13.8]	2774	20.1[17.4,23.1]	2787	34.8[31.7,38.1]
Men***	3859	11.4[9.7,13.3]	4771	44.3[42.1,46.6]	4784	24.2[22.3,26.2]	4761	48.4[45.8,51.0]	4767	41.3[38.9,43.6]
Poor	1296	14,0[11.5,16.9]	1573	33.6[30.3,37.0]	1581	33.2[30.2,36.3]	1571	75.8[72.3,78.9]	1565	48.6[45.2,52.0]
Middle	1588	11.7[9.0,15.0]	1973	49.9[46.5,53.4]	1978	22.6[19.8,25.7]	1967	35.8[32.6,39.0]	1972	37.1[33.5,40.8]
Upper	912	4.6[1.9,10.5]	1181	52.8[48.1,57.4]	1181	8.0[5.6,11.2]	1178	20.6[16.6,25.1]	1186	37.1[32.7,41.7]
Women***	5883	19.7[18.1,21.4]	7065	60.8[59.1,62.6]	7034	32.7[30.6,34.8]	7019	53.2[50.9,55.6]	7047	37.0[35.1,39.0]
Poor	2174	25.4[22.9,28.1]	2505	58.0[55.3,60.7]	2482	45.0[42.3,47.6]	2480	82.3[80.0,84.3]	2501	43.4[40.7,46.2]
Middle	2393	19.4[16.9,22.2]	2904	62.2[59.2,65.1]	2895	28.8[25.8,32.1]	2892	38.8[36.2,41.4]	2893	32.2[29.5,35.0]
Upper	1198	4.5[2.8,7.1]	1608	64.5[60.8,68.1]	1608	13.2[9.8,17.6]	1596	19.7[16.4,23.5]	1601	33.0[29.0,37.2]

***Significant at 0.1%.

#### HIV risk perception across socio-economic index groups

There was high personal HIV risk perception among the poor which declined significantly higher up in the socio-economic ladder. About, 40% among the poor, 26% among those in the middle SEI group and 10.9% of those in the upper SEI group believed that they were at high risk of HIV infection [P < 0.001] (Table 2). HIV risk perception was 4 times higher among the poor compared to the upper SEI. High HIV risk perception was reported more among women (32.7%) than men (24.3%) for all socio-economic groups (P < 0.001) although this difference was much pronounced among the poor (33.2% for men versus 45% for women) and the upper SEI group (8.0% for men and 13.2% for women) than it was for those in the middle (22.6% for men and 28.8% for men).

#### HIV-related stigma across socio-economic index groups

In general, 61.1% of the respondents had non-stigmatizing attitude (low-stigma score) towards HIV/AIDS, although women had better attitudes (63.0%) compared to men (58.7%) [P < 0.01]. A higher percentage of those in the poor SEI group had a high stigmatizing attitude towards HIV/AIDS (45.6%) compared to those in middle and upper SEI group, 34.4% and 34.8% respectively [P < 0.001].

#### HIV/AIDS information across socio-economic index groups

Only 20.5% of the poor SEI group had what could be labelled as “good access to HIV/AIDS information” compared to 79.9% in the upper SEI group [P < 0.001] (Table 2). Further, 74.8% of total respondents (63.6% for the poor and 82.9% for the upper SEI; P <0.001) listen to the radio and 76.7% watch television almost daily (49.2% for the poor and 95.5% for the upper SEI; P <0.001) (Additional file 1: Table S2). However, the frequencies of internet use and newspaper and magazine reading were much less although there was great disparity by socio-economic status. Only 41.2% read newspapers almost daily (23.4% among the poor SEI and 58% among the upper SEI groups; P < 0.001), 32.5% read magazines almost daily (19% among the poor SEI and 46% among the upper SEI groups [P < 0.001]; and 13.4% surfed internet almost daily [P < 0.001]) (Additional file 1: Table S2).

#### Sexual behaviour across the socio-economic index groups

Sexual risk behaviour was assessed using the reported number of sex partners (regular and non-regular), the number who reported that their recent sexual partner had other sexual partner(s) and the report of condom use during last sexual encounter. The survey did not enquire if respondents already knew their sero-status before anonymous HIV test was done although it is assumed that a good percentage of them were known HIV positives. However, 55.0% of HIV positive individuals reported to have had only one regular sex partner during the year preceding the survey compared to only 29.0% of those who were found to be HIV negatives (P < 0.001). Seventy percent of the HIV positives and 44.4% of the HIV negatives with high HIV risk perception believed that their sexual partner had other sex partner(s) (P < 0.001).

Only 2.8% of the total respondents reported that they had more than one regular sex partner during the last 12 months. There was a statistically significant but marginal difference between the poor (2.7%) and the upper SEI group (1.8%) [P < 0.001]. Similarly, only 4.8% of the respondents said that they had one or more non-regular sex partner during the past 12 months.

Majority of respondents (96.9%), across all the social-economic index groups, believed that condoms are easy to find at any time when one is in need to use. Women believed more so (98.0%) than men (95.0%) [P < 0.01].

#### Regression analysis results

Logistic regression results depicted in Tables 3 and 4 confirmed the statistical association indicated by the descriptive measures. Educational level was significantly associated with HIV risk perception, decreasing with increase in level of education, and this applies to all SEI groups (Table 3). Those with less than secondary school educational level perceived themselves to be at higher risk of acquiring HIV infection (OR = 1.46, p < 0.001) compared to those with tertiary level and above. Those in urban informal areas (mostly urban poor) had significantly increased the odds of high HIV prevalence compared to those in urban formal areas (mostly the urban non-poor) (OR = 2.74, P < 0.000, Table 3). The HIV risk perception is all high among those in urban informal (urban poor) compared to urban formal (non-poor) (OR = 2.34, p < 0.001, Table 3).

**Table 3:** Un-adjusted odds ratio for HIV prevalence (Model 1); HIV testing (Model 2), and risk perception (Model 3) and by background characteristics

	**Model 1: HIV prevalence**		**Model 2: HIV testing**		**Model 3: HIV risk perception**	
	**Un-adjusted odds ratio(OR)**	**Std. err**	**Un-adjusted odds ratio(OR)**	**Std. err**	**Un-adjusted odds ratio(OR)**	**Std. err**
**Sex:** Female vs. Male	1.91***	0.19	1.95***	0.11	1.52***	0.10
**Socio-economic Index** (Upper SEI^a^)						
Low (poor) SEI	5.48***	1.68	0.63***	0.05	5.46***	0.81
Middle SEI	3.97***	1.23			2.88***	0.40
**Race:** Blacks vs. Other Races	18.48***	3.10	0.72***	0.04	6.69***	0.70
**Location** (Urban Formal^a^)						
Urban Informal	2.74***	0.33			2.38***	0.28
Rural Informal	1.53***	0.19	0.65***	0.05	2.37***	0.22
Rural Formal					1.81***	0.27
**Education** (Secondary^a^)						
No schooling			0.29***	0.04	1.75***	0.26
Less Secondary	1.36***	0.16	0.48***	0.03	1.46***	0.13
Tertiary	0.38***	0.09	1.79***	0.21	0.57***	0.08
**Stigma Score**: High vs. Low	0.80***	0.07	0.65***	0.04	0.88**	0.06
**Information Access**: High vs. Low	0.56***	0.05	1.60***	0.10	0.47***	0.04
N	9,742		11730		11748	

^a^Reference group; ***significant at .1%; **significant at 1%.

**Table 4:** Adjusted odds ratio for HIV prevalence (Model M1), HIV testing (Model M2) and HIV risk (Model M3) perception within social economic groups by background characteristics

	**Model M1: HIV prevalence**		**Model M2: HIV testing**		**Model M3: HIV risk perception**	
	**Adjusted odds ratio (AOR)**	** Std. err**	**Adjusted odds ratio (AOR)**	** Std. err**	**Adjusted odds ratio (AOR)**	** Std. err**
**Poor SEI**	**(n = 3,190)**		**(n = 3,947)**		**(n = 3,933)**	
**Sex:** Female vs. Male	2.21***	0.30	2.81***	0.28	1.59***	0.14
**Race:** Blacks vs. Other Races	7.92***	2.38			2.74***	0.63
**Geographical Location** (Urban Formal^a^)						
Urban Informal						
Rural Informal	0.66**	0.12	0.75**	0.10		
Rural Formal					1.44**	0.27
**Education** (Secondary^a^)						
No schooling			0.35***	0.07		
Less secondary			0.61***	0.08		
Stigma Score: High vs. Low			0.76***	0.07	0.77***	0.07
Information Access: High vs. Low					0.75**	0.09
**Middle SEI**	**(n = 3,726)**		**(n = 4,753)**		**(n = 4,748)**	
**Sex:** Female vs. Male	1.66***	0.30	1.71***	0.17	1.33**	0.15
**Race:** Blacks vs. Other Races	10.42***	2.50	0.83*	0.09	4.10***	0.65
**Geographical Location** (Urban Formal^a^)						
Urban Informal	1.61**	0.30			1.43*	0.27
**Education** (Secondary^a^)						
No schooling			0.24***	0.07		
Less secondary			0.49***	0.05		
Tertially	0.54*	0.18	1.77***	0.31		
Stigma Score: High vs. Low	0.75*	0.12	0.76***	0.07		
Information Access: High vs. Low	0.76*	0.12			0.68***	0.08
**Upper SEI**	**(n = 1,926)**		**(n = 2,727)**		**(n = 2,728)**	
**Sex:** Female vs. Male			1.72***	0.24		
**Race:** Blacks vs. Other Races	49.02***	27.68			5.72***	1.42
**Geographical Location** (Urban Formal^a^)						
Urban Informal			2.87*	1.65		
**Education** (Secondary^a^)						
No schooling			0.23*	0.19		
Less secondary	2.10*	0.84	0.40***	0.07		
Tertially			2.26***	0.44	0.54*	0.19
Stigma Score: High vs. Low	0.42*	0.21	0.79*	0.11		

^a^Reference group; ***significant at .1%; **significant at 1% ; *significant at 5%.

Overall, the poor were 5 times more likely (OR = 5.46, p < 0.001; Table 3) to perceive themselves as being at high risk of acquiring HIV infection compared to those in the upper SEI group, while those in the middle SEI groups were 3 time more likely (OR = 2.88, p < 0.001) to have similar HIV risk perceptions . This is more pronounced among black Africans with odds of 6.69 compared to other races.

Among the poor SEI groups, being female (AOR = 2.21, P < 0.001) and black African (AOR = 7.92, P < 0.001) significantly increased the odds of high HIV prevalence (Table 4). As the black African population moves up in SEI, the odds of infection increase compared to other races. Being in the middle SEI group and living in urban informal settlement was significantly associated with high odds of HIV infection (AOR = 1.61, P < 0.001), 2-times higher than those living in formal urban areas.

Among the poor and middle SEI groups, being a black African female and living in rural formal (Tribal) or urban informal areas, increased the odds of perceiving self to be at risk of acquiring HIV infection (AOR = 1.4, p < 0.01; Table 4). Nonetheless, the poor were less likely to test for HIV infection (OR = 0.63, P < 0.001; Table 3) and this is more pronounced among black Africans compared to other races (OR = 0.72, p < 0.05; Table 3). Those with tertiary education were more likely to test across all socio-economic index groups. More females compared to men underwent HIV testing across all the SEI groups, but this was 3 times higher among the poor (AOR = 2.81, P < 0.001; Table 4). The odds of high stigma is roughly the same across all SEI groups (AOR = 0.76; p < 0.01; Table 4). For those in upper SEI group, the key factor for high HIV prevalence was being a black African (AOR = 49, P < 0.001, Table 4).

## Discussion

The findings showed high HIV prevalence among the poor in general and specifically among women, black African race and individuals with low educational status. The poor also felt more susceptible to HIV infection compared to those in upper SEI group. These study results are in agreement with current global thinking around the bidirectional relationship between socio-economic inequality and poor health outcome, in this case, HIV/AIDS [9,12,21-23]. In particular, the study points to the assumption that the poor in South Africa would have dual challenges of vulnerability (particularly women) and lack of opportunities to make better life choices due to limited education and HIV/AIDS services (such as information on and testing for HIV infection). Relative economic opportunities among black South Africans, referred to as “relative wealth” by Fox (2012), on the other hand were strongly associated high HIV prevalence. Magadi [24] observed similar results in her analysis of DHS survey data from 20 countries in sub-Saharan Africa in which the urban poor were noted to have significantly higher odds of HIV infection than their urban non-poor counterparts.

Some researchers argue that the “social history of AIDS and the way it was represented” in the early years gave a legacy to the continued stigma towards the disease [25] although it was believed to diminish in the era of ARV scaling [24]. Our analysis indicated a fairly high level of stigma at a time (in 2008) when nearly 40% of eligible South Africans were receiving ARV treatment [26]. More rigorous studies are indispensable to fully understand the reasons behind continued stigma and discrimination more than two decades into the epidemic and when living with HIV does not anymore mean “death sentence” as it used to be.

Stringer et al. [27] found that having had more than two lifetime sexual partners was a marker of high personal risk perception for HIV infection, although this perception did not predict HIV sero-status among women in Zambia. The survey used in this study did not enquire if respondents already knew their sero-status before anonymous HIV test was done although it is assumed that a good percentage of them were known HIV positives. It is plausible to assume that sexual risk perception is strongly linked to one’s sexual risk behaviour [28]. The extent to which this high risk perception serves as a motivational factor for adaptation of protective behaviour or remains subdued due to individuals’ socio-economic vulnerability needs to be further investigated.

In our analysis, the poor had limited access to HIV/AIDS information. A study on public communications and its role in reducing and eliminating health disparities by Viswanath (2006) indicated that those in low socioeconomic status (SES) also tend to gain less from the information flows than their counterparts of higher SES [29]. It is thus important to note that inequalities in access to mass media also follow the pattern of existing inequalities in HIV/AIDS service delivery and marginalize the poor and vulnerable.

One’s economic status created synergy with gender and level of educational attainment to significantly influence HIV-related outcomes in this study. This again is in agreement with many studies in sub-Saharan Africa which highlighted the disadvantage of being a woman living among communities with high socio-economic inequality [22,23]. Majority of people who did not test for HIV infection did so because of misconceptions about the disease which is closely related to their attitude towards the disease. The expansion of a routine and intensified campaign for HIV testing would contribute immensely towards breaking this link between misconceptions and stigma. Our analysis also highlighted the fact that perceived level of service quality may not significantly limit demand for HIV testing in public health facilities. Rather, poor access to these facilities could be seen to be an issue for poor people.

Finally, we believe that the socio-economic index that we constructed was able to discriminate inequalities by race, province, geographic location (urban and rural), level of income and educational attainments. The household asset or wealth status generated in this analysis was cut-down to three quintiles to minimize erroneous inferences based on extremely skewed socio-economic profiling. We also found instances of over-and-under estimation of poverty in some settings in a manner that is not reflective of the reality on the ground even after adjustment. These weaknesses mirror the usual critic on an asset index for its inability to clearly distinguish “poor households from the poorest ones”. Rustein (2008) suggests developing research instruments based on variables that appropriately describe economic situations both in the urban and rural area and adequately discriminate different economic groups among residents and calculating a composite index of the two.

## Conclusions

Using a simplified socio-economic index profile, this study was able to underline the disproportionate distribution of HIV disease burden and fear among the poor in South Africa. The poor were further disadvantaged by lack of access to HIV information and HIV/AIDS services such as testing for HIV infection. Our socio-economic index profiling could not make a clear discrimination within the “middle class and wealthy” mainly because of weaknesses in measures of living standards. There is a compelling urgency for the national HIV/AIDS response to maximize program focus for the poor particularly women.

## Endnotes

^a^Study activities were approved by the Human Science’s Research Council’s Research Ethics Committee (REC 2/13/10/07) and Human Subjects Review (IRB # 00006347) from the Centre for Disease Control and Prevention’s Global AIDS Programme.

^b^Weighting of the sample by age, race group and province was applied to ensure the study estimates are representative of the general population.

## Competing interests

Both authors declare that they have no competing interests.

## Authors’ contributions

NW, NT conceived of the study, and participated in its design and coordination. NW, NT wrote the draft manuscript. NW, NT performed the statistical analysis. Both authors read and approved the final manuscript.

## Pre-publication history

The pre-publication history for this paper can be accessed here:

http://www.biomedcentral.com/1471-2458/13/1037/prepub

## Supplementary Material

Additional file 1 **MCA Weights and Variance of the Variable modalities as Table S1 and Sources of HIV/AIDS information by Socio-economic index as Table S2.** Click here for file
