# Supplementary material for: Socio-economic inequality and HIV in South Africa
Source: BMC Public Health. 2013 Nov 4;13:1037. doi: 10.1186/1471-2458-13-1037 (PMC4228412; doi:10.1186/1471-2458-13-1037)
Supplement: Additional file 1 — MCA Weights and Variance of the Variable modalities as Table S1 and Sources of HIV/AIDS information by Socio-economic index as Table S2. [file 1471-2458-13-1037-S1.docx]

Additional file 1: Table S1: MCA Weights and variance of the variable modalities/Categories

| Variable |  |  |  |
| --- | --- | --- | --- |
| ***Source of drinking water*** | Mean | Std. Dev. | MCA Weights |
| Piped Tap water in Dwelling | 0.651 | 0.477 | 0.672 |
| Piped Tap water in Yard | 0.173 | 0.379 | -0.357 |
| Community Tap | 0.108 | 0.31 | -2.001 |
| Water Tanker | 0.013 | 0.115 | -2.086 |
| Well or Spring | 0.015 | 0.12 | -2.481 |
| Stream or River | 0.026 | 0.158 | -2.999 |
| Rain Water Tank | 0.003 | 0.059 | -1.9 |
| Dam | 0.002 | 0.042 | -2.936 |
| Other | 0.009 | 0.093 | -1.598 |
| ***Source of cooking energy*** |  |  |  |
| ***Electricity*** | ***0.784*** | ***0.412*** | ***0.577*** |
| Coal | 0.017 | 0.13 | -1.063 |
| Wood | 0.109 | 0.312 | -2.424 |
| Gas | 0.027 | 0.163 | -0.274 |
| Paraffin | 0.06 | 0.238 | -2.661 |
| Other | 0.002 | 0.046 | -2.735 |
| ***Type of Toilet Facility*** |  |  |  |
| ***Flush Toilet*** | ***0.679*** | ***0.467*** | ***0.71*** |
| Pit Toilet | 0.251 | 0.433 | -1.191 |
| Chemical or Bucket Toilet | 0.021 | 0.142 | -1.728 |
| Other toilet | 0.006 | 0.074 | -2.116 |
| No toilet | 0.045 | 0.206 | -3.19 |
| ***Other assets*** |  |  |  |
| Access to Electricity | 0.878 | 0.327 | 0.426 |
| No Access to electricity | 0.122 |  | -3.061 |
| Working Refrigerator | 0.782 | 0.413 | 0.608 |
| No Working Refrigerator | 0.218 |  | -2.206 |
| Working radio | 0.822 | 0.383 | 0.333 |
| No working Radio | 0.178 |  | -1.552 |
| Working TV | 0.801 | 0.399 | 0.555 |
| No Working TV | 0.199 |  | -2.266 |
| Working Telephone | 0.311 | 0.463 | 1.067 |
| No Working telephone | 0.689 |  | -0.484 |
| Working Cell phone | 0.862 | 0.345 | 0.222 |
| NO Working Cell Phone | 0.138 |  | -1.394 |

**Additional file 1: Table S2: Sources of good HIV/AIDS information by Socio- economic index**

|  | **Socio-Economic Index Groups** | | | | | | | | |
| --- | --- | --- | --- | --- | --- | --- | --- | --- | --- |
|  | **Poor** |  | **Middle** |  | **Upper** |  | **Total** |  |  |
|  | **%** | **95% CI** | **%** | **95% CI** | **%** | **95% CI** | **%** | **95% CI** | **n** |
| **Information Access** |  |  |  |  |  |  |  |  |  |
| Low | 79.5 | [77.5-81.5] | 37.4 | [35.3-39.6] | 20.1 | [17.4-23.1] | 50.9 | [48.8-53.0] | 5663 |
| High | 20.5 | [18.5-22.5] | 62.6 | [60.4-64.7] | 79.9 | [76.9-82.6] | 49.1 | [47.0-51.2] | 6021 |
|  |  |  |  |  |  |  |  |  |  |
| **Listening to radio** |  |  |  |  |  |  |  |  |  |
| Once/Never | 36.4 | [34.1-38.8] | 20.2 | [18.6-21.8] | 17.1 | [14.5-20.1] | 26 | [24.8-27.4] | 3111 |
| Almost daily | 63.6 | [61.2-65.9] | 79.8 | [78.2-81.4] | 82.9 | [79.9-85.5] | 74 | [72.6-75.2] | 8679 |
|  |  |  |  |  |  |  |  |  |  |
| **Watching Television** |  |  |  |  |  |  |  |  |  |
| Once/Never | 50.8 | [47.6-54.0] | 5.8 | [4.9-7.0] | 4.5 | [3.4-5.9] | 23.3 | [21.4-25.4] | 2548 |
| Almost daily | 49.2 | [46.0-52.4] | 94.2 | [93.0-95.1] | 95.5 | [94.1-96.6] | 76.7 | [74.6-78.6] | 9242 |
|  |  |  |  |  |  |  |  |  |  |
| **Read a Magazine** |  |  |  |  |  |  |  |  |  |
| Once/Never | 81 | [79.2-82.8] | 60.6 | [58.1-63.0] | 53.8 | [50.3-57.2] | 67.5 | [65.8-69.1] | 7714 |
| Almost daily | 19 | [17.2-20.8] | 39.4 | [37.0-41.9] | 46.2 | [42.8-49.7] | 32.5 | [30.9-34.2] | 4047 |
|  |  |  |  |  |  |  |  |  |  |
| **Read a Newspaper** |  |  |  |  |  |  |  |  |  |
| Once/Never | 76.6 | [74.4-78.7] | 49.3 | [46.8-51.8] | 42 | [38.3-45.9] | 58.8 | [56.8-60.7] | 6786 |
| Almost daily | 23.4 | [21.3-25.6] | 50.7 | [48.2-53.2] | 58 | [54.1-61.7] | 41.2 | [39.3-43.2] | 4986 |
|  |  |  |  |  |  |  |  |  |  |
| **Surf the Internet** |  |  |  |  |  |  |  |  |  |
| Once/Never | 98.2 | [97.6-98.7] | 87.4 | [85.7-89.0] | 58.8 | [54.9-62.6] | 86.6 | [85.2-88.0] | 9979 |
| Almost daily | 1.8 | [1.3-2.4] | 12.6 | [11.0-14.3] | 41.2 | [37.4-45.1] | 13.4 | [12.0-14.8] | 1776 |
